# Supplementary material for: Assessment of In Vitro Bioaccessibility and In Vivo Oral Bioavailability as Complementary Tools to Better Understand the Effect of Cooking on Methylmercury, Arsenic, and Selenium in Tuna
Source: Toxics. 2021 Feb 3;9(2):27. doi: 10.3390/toxics9020027 (PMC7913187; doi:10.3390/toxics9020027)
Supplement: Supplementary file 1 [file toxics-09-00027-s001.zip › toxics-1084049 supplementary 1.docx]

Supplementary Materials: Assessment of In Vitro Bioaccessibility and In Vivo Oral Bioavailability as Complementary Tools to Better Understand the Effect of Cooking on Methylmercury, Arsenic, and Selenium in Tuna

Tania Charette, Danyel Bueno Dalto, Maikel Rosabal, J. Jacques Matte and Marc Amyot

**Figure S1.** Venous RBC MeHg concentration (µg/L) of five individuals within 540 minutes following consumption of a single raw (1R, 2 and 3) and cooked tuna (1C, 4 and 5) meal. Preconsumption concentration was subtracted from each presented value. Values were different from zero (Time effect, *p* < 0.05), were not influenced by treatments (Treatment effect, *p* > 0.05) and no interaction between time and treatments (Time effect × treatment, *p* > 0.05) was measured.


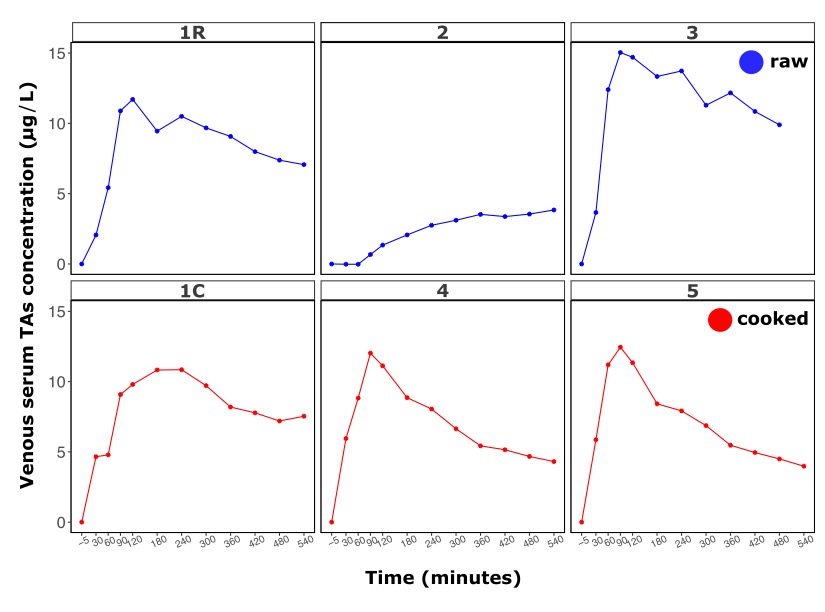


**Figure S2.** Venous serum TAs concentration (µg/L) of five individuals within 540 minutes following consumption of a single raw (1R, 2 and 3) and cooked (1C, 4 and 5) tuna meal. Preconsumption concentration was subtracted from each presented value. Values from raw treatment as function of time were different from zero (Time effect, *p* < 0.05), in the contrary of cooked treatment data (Time effect, *p* > 0.05). Treatment influenced the values as function of time (Time effect $\times$ treatment, *p* < 0.05).

**Figure S3.** Venous serum TAs and AsB concentration (µg/L) as function of time and treatment of one individual blood profile (1 single pig) within 540 minutes following consumption of a single raw (1R) and cooked (1C) tuna meal. Raw and cooked meal were spaced by one week. Preconsumption concentration was subtracted from TAs presented value. AsB preconsumption value was not detectable.


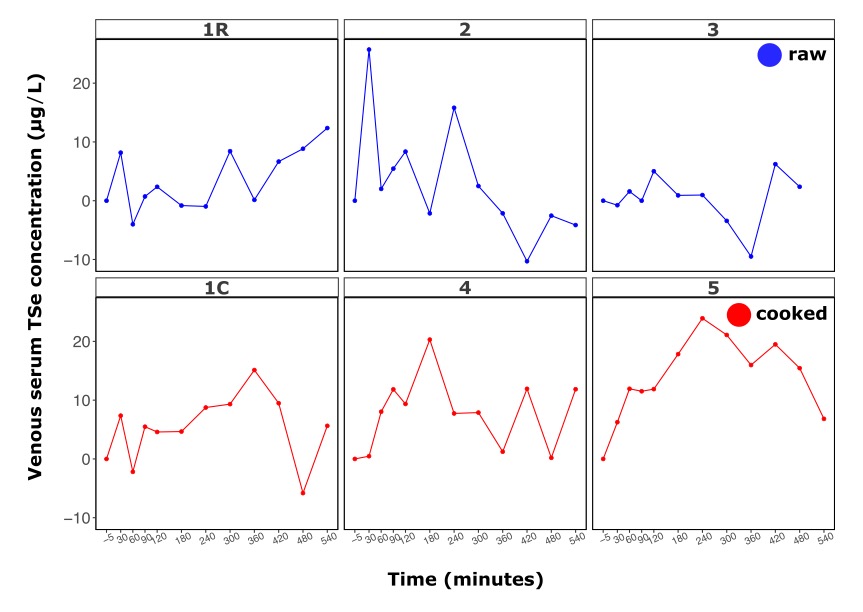


**Figure S4.** Venous serum TSe concentration (µg/L) of five individuals within 540 minutes following consumption of a single raw (1R, 2 and 3) and cooked (1C, 4 and 5) tuna meal. Preconsumption concentration was subtracted from each presented value. Values were not different from zero (Time effect, *p* > 0.05) and were not influenced by treatments (Time effect $\times$ treatment, *p* > 0.05).

**Figure S5.** Mean (± SD) proportion (%) of the total quantity of different elements distributed between RBCs and the serum fraction over 540 min postprandial (*n* = 34 and 35 for the raw tuna meal, and *n* = 36 for the cooked tuna meal treatment). Total quantity of each blood compartment was adjusted to their respective volumes (RBC concentration was adjusted as a function of hematocrit ([M_RBC_] × blood volume × hematocrit) and serum concentration as a function of serum volume ([M_serum_] × blood volume × (1 − hematocrit)). * indicates that mean proportion varies as a function of time (time effect, *p* < 0.05). No difference was measured between the raw and cooked treatments (treatment effect, *p* ˃ 0.05).

**Table S1.** Element analyzed, their detection limit and technology used.

| **Element** | **Detection Limit** | **Unity** | **Technology/Instrument** |
| --- | --- | --- | --- |
| THg | 0.05 | ng/g | DMA 80 |
| THg | 0.04 | ng/L | CVAFS, Tekran 2600 |
| MeHg | 0.01 | ng/L | GC-CVAFS, Tekran 2700 |
| Se | 0.49 | ng/L | ICP-MS/MS 8900 Triple Quadrupole, Agilent |
| Na | 100 |  |  |
| Mg | 60 |  |  |
| K | 100 |  |  |
| Mn | 2 |  |  |
| Fe | 100 |  |  |
| Cu | 2 |  |  |
| Zn | 100 |  |  |
| V | 2 |  |  |
| Sr | 2.5 |  |  |
| Ba | 0.5 |  |  |
| TAs | 0.71 |  |  |
| AsB | 0.01 |  |  |
| DMA | 0.012 |  |  |
| MMA | 0.008 |  |  |
| As^5+^ | 0.01 |  |  |
| As^3+^ | 0.012 |  |  |

**Table S2.** Details of the dose of TAs ingested and TAs changes in pigs’ serum.

|  | **Raw** | | | **Cooked** | | |
| --- | --- | --- | --- | --- | --- | --- |
|  | **1R*** | **2** | **3** | **1C*** | **4** | **5** |
| **Preconsumption values (µg/L)** | 2.4 | 1.7 | 2.1 | 1.3 | 1.4 | 0 |
| **AUC (0-540) (µg x min/L/µg)** | 2.5 | 0.7 | 3.2 | 2.3 | 2.0 | 2.5 |
| **Cmax (t0 corrected) (µg/L)** | 11.7 | 3.8 | 15.0 | 10.9 | 12.0 | 12.5 |
| **Tmax (min)** | 120 | NA^†^ | 90 | 240 | 90 | 90 |
| **Intake (µg)** | 499 | 500 | 528 | 657 | 635 | 503 |
| **Dose (µg/kg bw)** | 9.6 | 8.7 | 10.2 | 12.6 | 10.9 | 7.7 |

* Pig 1 received both cooked and raw treatment a week apart. ^†^ Pig did not reach Tmax.

**Table S3.** Ratio of elements distribution between blood compartments in this study and from the literature. Values presented are averages and standard deviations (SD).

| **Elements** | **Medium of Exposure** | **Model** | ***n*** | **WB: serum ratio** | **RBC: Serum Ratio or Range of Values** | **References** |
| --- | --- | --- | --- | --- | --- | --- |
| **THg** | 1 fish meal (raw) | Pig | 34 * | 4.7 (1.8) | 12.9 (5.8) | This study |
|  | 1 fish meal (cooked) | Pig | 36 * | 4.7 (2.6) | 13.1 (8.3) | This study |
|  | 1 fish meal (cooked) | Human | 7 |  | [20;22] ** | Kershaw et al., 1980 |
|  | Chronic exposure through fish consumption | Human (eldery) | 1000 | 2.2 (1.4) |  | Schultze et al., 2014 |
|  | Chronic exposure through fish consumption | Human (ado) | 335 | 2.5 (0.92) |  | Barany et al., 2002 |
|  | Chronic exposure through fish consumption | Human | 206 |  | [2;13] ** | Skerfving, 1974 |
|  | Chronic exposure through fish consumption | Human | 79 |  | estimated by the author at 4 ** | Svensson et al., 1992 |
|  | Chronic exposure through fish consumption | Women (postpartum) | 9 | 2.1 (0.7) ** |  | Suzuki et al., 1971 |
|  | Chronic exposure through fish consumption | Human | 27 | 4.8 (0.8) ** | 9.6 (0.8) ** | Yaginuma-Sakurai et al., 2012 |
| **MeHg** | 1 fish meal (raw) | Pig | 34 * | 4.09 (1.1) | 11.01 (3.6) | This study |
|  | 1 fish meal (cooked) | Pig | 36 * | 5.26 (1.2) | 14.7 (3.6) | This study |
|  | 1 single oral dosage of 15 mg/kg bw | Rat | 4 |  | 180 ** | Clausing et al., 1984 |
|  | 1 single oral dosage of 15 mg/kg bw | Quail | 4 |  | 58 ** | Clausing et al., 1984 |
| **TAs** | 1 fish meal (raw) | Pig | 34 * | 0.8 (0.1) | 0.25 (0.2) | This study |
|  | 1 fish meal (cooked) | Pig | 36 * | 0.8 (0.1) | 0.39 (0.3) | This study |
| **TSe** | 1 fish meal (raw) | Pig | 34 * | 1.3 (0.3) | 1.9 (0.8) | This study |
|  | 1 fish meal (cooked) | Pig | 36 * | 1.3 (0.2) | 1.9 (0.6) | This study |
|  | Chronic exposure through fish consumption | Human (ado) | 243 | 1.0 (0.02) |  | Barany et al., 2002 |
| **Cu** | 1 fish meal (raw) | Pig | 34 * | 0.8 (0.1) | 0.4 (0.2) | This study |
|  | 1 fish meal (cooked) | Pig | 36 * | 0.7 (0.1) | 0.1 (0.2) | This study |
|  | Chronic exposure through fish consumption | Human (eldery) | 1000 | 0.9 (0.3) |  | Schultze et al., 2014 |
|  | Chronic exposure through fish consumption | Human (ado) | 342 | 0.9 (0.3) |  | Barany et al., 2002 |
|  | Normal life exposure | Normal humans (not sick) | 106 |  | 0.8 ** | Herring et al., 1960 |
| **Zn** | 1 fish meal (raw) | Pig | 34 * | 2.9 (0.5) | 6.9 (1.9) | This study |
|  | 1 fish meal (cooked) | Pig | 36 * | 2.4 (0.4) | 5.6 (1.4) | This study |
|  | Chronic exposure through fish consumption | Human (eldery) | 1000 | 8.6 (0.3) |  | Schultze et al., 2014 |
|  | Chronic exposure through fish consumption | Human (ado) | 336 | 6.1 (0.2) |  | Barany et al., 2002 |
|  | Normal life exposure | Normal humans (not sick) | 106 |  | 3.3 ** | Herring et al., 1960 |
| **Mn** | 1 fish meal (raw) | Pig | 34 * | 2.5 (1.2) | 5.6 (4.1) | This study |
|  | 1 fish meal (cooked) | Pig | 36 * | 1.9 (1) | 3.9 (2.8) | This study |
|  | Chronic exposure through fish consumption | Human (eldery) | 1000 | 5.0 (1.0) |  | Schultze et al., 2014 |
| **Na** | 1 fish meal (raw) | Pig | 34 * | 0.7 (0.1) | 0.1 (0.1) | This study |
|  | 1 fish meal (cooked) | Pig | 36 * | 0.6 (0.1) | 0.1 (0.1) | This study |
|  | Normal life exposure | Normal humans (not sick) | 50 |  | 0.1 (0.2) ** | Valberg et al., 1965 |
| **Mg** | 1 fish meal (raw) | Pig | 34 * | 2.1 (0.2) | 4.5 (0.6) | This study |
|  | 1 fish meal (cooked) | Pig | 36 * | 1.7 (0.3) | 3.4 (1.0) | This study |
|  | Normal life exposure | Normal humans (not sick) | 106 |  | 3.6 ** | Herring et al., 1960 |
|  | Normal life exposure | Normal humans (not sick) | 50 |  | 3.7 (0.3) ** | Valberg et al., 1965 |
| **K** | 1 fish meal (raw) | Pig | 34 * | 10.8 (1.8) | 32.7 (5.6) | This study |
|  | 1 fish meal (cooked) | Pig | 36 * | 9.7 (1.6) | 28.9 (5.0) | This study |
|  | Normal life exposure | Normal humans (not sick) | 50 |  | 24.9 (0.2) ** | Valberg et al., 1965 |
| **Fe** | 1 fish meal (raw) | Pig | 34 * | 292.7 (92.2) | 943.4 (281.4) | This study |
|  | 1 fish meal (cooked) | Pig | 36 * | 230.8 (98.3) | 731.3 (275.0) | This study |
| **V** | 1 fish meal (raw) | Pig | 34 * | 0.7 (0.1) | 0.1 (0.1) | This study |
|  | 1 fish meal (cooked) | Pig | 36 * | 0.7 (0.1) | 0.1 (0.2) | This study |
| **Sr** | 1 fish meal (raw) | Pig | 34 * | 0.8 (0.2) | 0..5 (0.5) | This study |
|  | 1 fish meal (cooked) | Pig | 36 * | 0.7 (0.1) | 0.1 (0.3) | This study |
| **Ba** | 1 fish meal (raw) | Pig | 34 * | 2.4 (1.9) | 5.4 (6.2) | This study |
|  | 1 fish meal (cooked) | Pig | 36 * | 1.3 (0.7) | 1.9 (2.3) | This study |
